# Predicting COVID-19 severity in pediatric patients using machine learning: a comparative analysis of algorithms and ensemble methods

**DOI:** 10.1038/s41598-025-15366-1

**Published:** 2025-08-08

**Authors:** Babak Pourakbari, Setareh Mamishi, Sepideh Keshavarz Valian, Shima Mahmoudi, Reihaneh Hosseinpour Sadeghi, Mohammad Reza Abdolsalehi, Mahmoud Khodabandeh, Mohammad Farahmand

**Affiliations:** 1 Pediatric Infectious Disease Research Center, Tehran University of Medical Sciences, Tehran, Iran; 2 Department of Infectious Diseases, Pediatrics Center of Excellence, Children’s Medical Center, Tehran University of Medical Sciences, Tehran, Iran; 3 School of Medicine, Tehran University of Medical Sciences, Tehran, Iran; 4 Biotechnology Centre, Silesian University of Technology, Gliwice, 44-100 Poland

**Keywords:** COVID-19, Pediatric patients, Machine learning, Severity prediction, Random forest, Ensemble model, Machine learning, Predictive medicine, Computational biology and bioinformatics, Diseases

## Abstract

COVID-19 has posed a significant global health challenge, affecting individuals across all age groups. While extensive research has focused on adults, pediatric patients exhibit distinct clinical characteristics that necessitate specialized predictive models for disease severity. Machine learning offers a powerful approach to analyzing complex datasets and predicting outcomes, yet its application in pediatric COVID-19 remains limited. This study evaluates the performance of machine learning algorithms in predicting disease severity among pediatrics. A retrospective analysis was conducted on a dataset of 588 pediatric with confirmed COVID-19, incorporating demographic, clinical, and laboratory variables. Various machine learning models were trained and assessed, with a SuperLearner ensemble model implemented to enhance predictive accuracy. Among the models, Random Forest exhibited the highest performance, achieving an accuracy of 90.1%, sensitivity of 90.2%, and specificity of 90.1%. The SuperLearner ensemble further improved predictive performance, demonstrating the lowest mean risk estimate. Key predictors, including oxygen saturation, respiratory parameters, and specific laboratory markers, played a crucial role in distinguishing severe from non-severe cases. These findings emphasize the potential of machine learning, particularly ensemble methods, in improving risk stratification for pediatric COVID-19. Integrating these predictive models into clinical practice could support early identification of high-risk patients and optimize clinical decision-making.

**Supplementary Information:**

The online version contains supplementary material available at 10.1038/s41598-025-15366-1.

## Introduction

In December 2019, the first cases of the new coronavirus disease (COVID-19) were reported^1^. Since then, numerous global waves of infection have emerged^2^. In early 2022, the Omicron variant replaced previous variants and quickly spread worldwide due to its high transmission rate and immune evasion capacity. Numerous Omicron subvariants have since emerged, further complicating efforts to control the virus^3^.

Despite the implementation of comprehensive preventive measures, COVID-19 has effectively spread around the world. This persistent nature can be attributed to several factors, including the emergence of new variants, failures in public compliance with preventative measures, and the worldwide interconnectivity that facilitates the circulation and transmission of the virus. Even with widespread vaccination efforts, COVID-19 remains a significant global health challenge, with new outbreaks and cases reported regularly^4^.

COVID-19 presents a wide spectrum of clinical manifestations, ranging from asymptomatic cases to mild or moderate symptoms, and in some instances, progressing to severe, life-threatening disease^2,5,6^. While adults served as the main and primary subject of several research studies, children were also considered as a susceptible group that can also experience severe COVID-19 outcomes, however with a lower frequency^7^. So, understanding the characteristics and factors that contribute to severe manifestations of COVID-19 in pediatric patients is essential for effective and timely intervention, as well as implementing effective treatment strategies.

Advanced machine learning (ML) algorithms provide a robust method for forecasting disease outcomes by the analysis of complex datasets. Additionally, these algorithms have the capacity to identify patterns and relationships among variables that may not be readily apparent through conventional statistical methods. In the context of COVID-19, ML models have been employed for predicting patient outcomes, resource requirements, and disease progression by analyzing a variety of data inputs, including demographic, clinical, and laboratory characteristics^8^.

Several studies have applied ML techniques in clinical diagnostics, particularly in cardiovascular disease and arrhythmia. Table 1 summarizes key findings and limitations of these prior works. Recent studies have demonstrated the utility of machine learning for clinical risk prediction, particularly in domains such as cardiovascular and arrhythmia classification. For example, Sharma et al.^9^ compared heterogeneous ML algorithms for arrhythmia detection, integrating feature selection and reporting improved performance across classifiers such as Random Forest and XGBoost. Similarly, Dhanka et al.^10^ evaluated logistic regression and XGBoost for coronary artery heart disease diagnosis, emphasizing the value of hyperparameter tuning in improving accuracy. Other studies have employed hybrid strategies, such as combining particle swarm optimization (PSO) with classifiers to enhance predictive performance in arrhythmia classification^11^ or applying genetic algorithms to optimize model performance in heart disease prediction^12^. These approaches report high accuracy but primarily focus on adult populations and lack ensemble interpretability frameworks.

Moreover, a comprehensive review by Sharma et al.^13^ highlighted persistent limitations in existing ML-based diagnostic systems, including class imbalance, insufficient external validation, and limited integration of explainable AI. Building on these observations, several recent studies such as those by Tofighi et al.^14^, Campagnini et al.^15^, Doudesis et al.^16^, Pathan and Imran^17^ and additional studies^18–26^ further emphasize the underrepresentation of pediatric populations, inadequate external validation, and the lack of interpretable modeling across various clinical contexts. Despite these methodological advancements, there remains a critical need for models that combine ensemble learning with transparency, especially in pediatric care. Addressing this gap, the present study employs a diverse set of interpretable models within a SuperLearner ensemble framework to predict COVID-19 severity in pediatric patients.

**Table 1:** Summary of existing ML studies and identified gaps.

Study	ML Methods	Domain/dataset	Key findings	Research gap
Sharma et al.^9^	RF, LDA, GNB, DT, XGB, PSO	Arrhythmia (UCI dataset)	Hybrid models with PSO improved accuracy to 95.24%	No pediatric focus; limited ensemble comparison
Dhanka et al.^10^	Logistic regression, XGBoost	Coronary artery disease (Statlog)	Optimized XGBoost achieved 91.85% accuracy	Only binary classification; limited ensemble use
Tofighi et al.^14^	GBM, DRF, LR, DL	Acute STEMI (Tehran)	DRF and GBM models achieved AUC of 0.92 and 0.91 for MACE prediction	Limited focus on pediatric populations; risks in diverse demographics
Dhanka & Maini^11^	PSO + XGBoost	Cardiac arrhythmia (UCI)	F1 score 96.3%; low computational cost	No interpretability or pediatric stratification
Campagnini et al.^15^	Various ML algorithms	Post-stroke rehabilitation	Identified common predictors; highlighted methodological limitations	Small sample sizes; limited external validation; high heterogeneity
Kumar et al.^12^	GA + Multiple ML models	Statlog + Cleveland HD	GA-XGB model reached 94.83% accuracy	Adult focus only; ensemble learning not explored
Doudesis et al.^16^	ML Models integrated with clinical features	Myocardial infarction (10,038 patients)	CoDE-ACS score achieved AUC of 0.953; improved low/high probability classification	No pediatric focus; limited integration with EKG data
Dhanka & Maini (2024)	XGBoost + Optuna	Cleveland HD	Accuracy of 95.45% with outlier handling	No external validation; no pediatric focus
Pathan and Imran^17^	Logistic Regression, Random Forest, SVM, KNN, Decision Tree, Gradient Boosting, LSTM	Cardiovascular diseases	Gradient Boosting and LSTM achieved high accuracy and ROC AUC scores	No pediatric focus; model interpretability not addressed
Sharma et al.^9^	Systematic review	Various HD datasets	Identified issues: data heterogeneity, lack of interpretability	No pediatric focus; limited real-world deployment

Given the growing body of research on the use of machine learning to predict COVID-19 outcomes, there remains a substantial gap in studies that focus on pediatric populations^27^. Most existing models are designed and validated on adult cohorts, often neglecting the unique clinical and immunological characteristics of children, such as different symptom profiles, disease trajectories, and the incidence of pediatric-specific conditions like multisystem inflammatory syndrome in children (MIS-C)^28–30^. There is a pressing need for child-specific models that leverage clinical and laboratory markers to accurately stratify disease severity.

To address this gap, the present study aims to:

- Evaluate the performance of various machine learning algorithms in predicting COVID-19 severity in pediatric patients.
- Compare individual models and an ensemble SuperLearner model to identify the most accurate and robust approach.
- Identify key clinical, demographic, and laboratory predictors associated with severe outcomes in children.
- Contribute to the development of specialized decision support tools for pediatric care settings.

Recent studies have demonstrated the promise of machine learning models in predicting COVID-19 severity and prognosis in adult populations, with a growing emphasis on explainability. For example, Chadaga et al.^31^ utilized multiple explainable AI approaches for COVID-19 diagnosis using clinical markers in Ecuador. Similarly, another study developed a multi-class framework for prognosis prediction^32^ while others have explored differential diagnosis using interpretable models. Notably, Chadaga et al.^33^ proposed a stacked ensemble learning approach for severity prediction based on clinical biomarkers. These studies underscore the effectiveness of combining machine learning with explainability to improve clinical trust and usability. However, these models were not tailored for children, who differ significantly in disease manifestation and immune response. This study builds upon this work by adapting such methods to the pediatric setting, where dedicated models are critically needed.

This study aims to fill this gap by comparing different ML algorithms using a comprehensive dataset that includes demographic, clinical, and laboratory variables specific to children. The goal is to identify effective models for early risk stratification to support improved clinical decision-making and resource allocation.

While previous studies have investigated COVID-19 severity prediction using machine learning, most have centered on adult populations or relied on single-model approaches. Building on this foundation, our work applies an ensemble of interpretable machine learning models specifically the SuperLearner framework to pediatric patients, thereby addressing an important gap in the literature.

This study is motivated by the limited availability of pediatric-specific machine learning models for COVID-19 severity prediction, the underutilization of ensemble techniques in this context, and the need for interpretable and clinically actionable tools. The core contributions of this work are as follows:

- Development of a pediatric-focused severity prediction model using curated clinical and laboratory features.
- Implementation of multiple machine learning classifiers and a SuperLearner ensemble framework to compare performance.
- Integration of explainable AI techniques, including permutation importance and SHAP, to interpret model behavior.
- Comprehensive evaluation using multiple performance metrics and external hold-out validation.

## Methods

### Dataset description

The present study, conducted a retrospective analysis on a hospital-based registry database for COVID-19, from February 25, 2020, to November 15, 2021. The dataset consisted of patient data obtained from Children Hospital Medical Center, a specialized pediatric referral institution in Tehran, Iran. The sampling protocol received approval from the Ethics Committee of Tehran University of Medical Sciences, Tehran, Iran (IR.TUMS.CHMC.REC1399.069). All methods were performed in accordance with the relevant guidelines and regulations, including the Declaration of Helsinki. Informed consent was obtained from all participants and/or their legal guardians prior to data collection.

The registry database included 93 primary features (Supplementary Table 1) categorized into 4 main classes: demographics (3 features), clinical characteristics (40 features), comorbidity history (6 features), laboratory results (43 features) and an outcome variable indicating severity status (0: Non-severe, 1: Severe). Quantitative parameters were measured numerically, while nominal parameters were recorded as binary responses (1: Yes or 0: No). Supplementary Table 2 describes dataset structure and class distribution.

Severe COVID-19 was defined based on objective clinical outcomes adapted from the World Health Organization (WHO) COVID-19 clinical progression scale^34^with severe disease classified by the occurrence of one or more of the following critical endpoints: ICU admission for close monitoring or advanced support, intubation for respiratory failure, mechanical ventilation for respiratory support, and death attributable to COVID-19 complications. These criteria were chosen to reflect clinically significant disease progression and align with prior peer-reviewed studies^35–37^. This severity classification was used as the outcome variable (Y) in the supervised learning models, where the goal was to distinguish between severe and non-severe cases based on the predictor variables (Supplementary Table 3).

Each patient was registered only once in the dataset, with only the first set of laboratory results collected during their initial admission being considered. Demographic and comorbidity history information was sourced from medical records or directly from patients and their companions. Clinical features were documented upon admission, and laboratory tests were conducted during the initial hours of hospitalization. Patients were initially assessed in the hospital’s emergency department before being admitted and subsequently transferred to the appropriate hospital wards. Laboratory tests were conducted during the first hours of their hospital stay, and the results were integrated into their medical records. Finally, Laboratory results were integrated into the patients’ medical records.

Chest CT imaging was conducted only when requested by a specialist. The identified CT features of COVID-19 included ground-glass opacities, consolidations, opacity distribution, pleural effusion, fibrosis, and nodules. Additionally, the extent of bilateral lung involvement and the number of affected lobes were evaluated. All CT images were independently reviewed by two radiologists to ensure accuracy. Any discrepancies between the initial reviews were resolved through consultation with a senior radiologist.

### Data pre‑processing

In the present study, data pre-processing was undertaken prior to training the machine learning algorithms in order to addressing irrelevant and unreliable data. Initially, records with more than 60% missing data were excluded from the dataset. For the remaining records, missing values in binary variables were imputed using logistic regression, while missing values in continuous variables were imputed using predictive mean matching using ‘mice’ package^38^ in R environment. All continuous variables were standardized using Z-score normalization to ensure consistency across features. This transformation was performed using the ‘step_normalize’ function from the ‘recipes’ package and was applied after multiple imputation to maintain data integrity.

As previously indicated, the present study uses chest CT scores to show each patient’s level of pulmonary involvement. Initially, two-digit codes were used to reflect different CT findings, such as ground-glass opacities, consolidation, bilateral lung disease, more than two lobes affected, pulmonary nodule, pleural effusion, lymphadenopathy, empyema, and fibrosis. Each variable was coded as 0 (No) or 1 (Yes). To generate a composite score that exhibited the overall degree of pulmonary involvement, the binary chest CT score values were combined into a single score ranging from 0 to 9 by summing the individual binary values for each patient.

Also, to properly include categorical variables such as blood group in the machine learning models, the blood group variable was transformed into dummy variables. This approach allows the model to handle categorical data more effectively by converting each category into a binary variable. The ‘fastDummies’ package^39^ was used to perform this transformation. This transformation ensured that the categorical information was appropriately represented in the model, improving its ability to capture the relationship between blood group and COVID-19 severity.

**Fig. 1:**
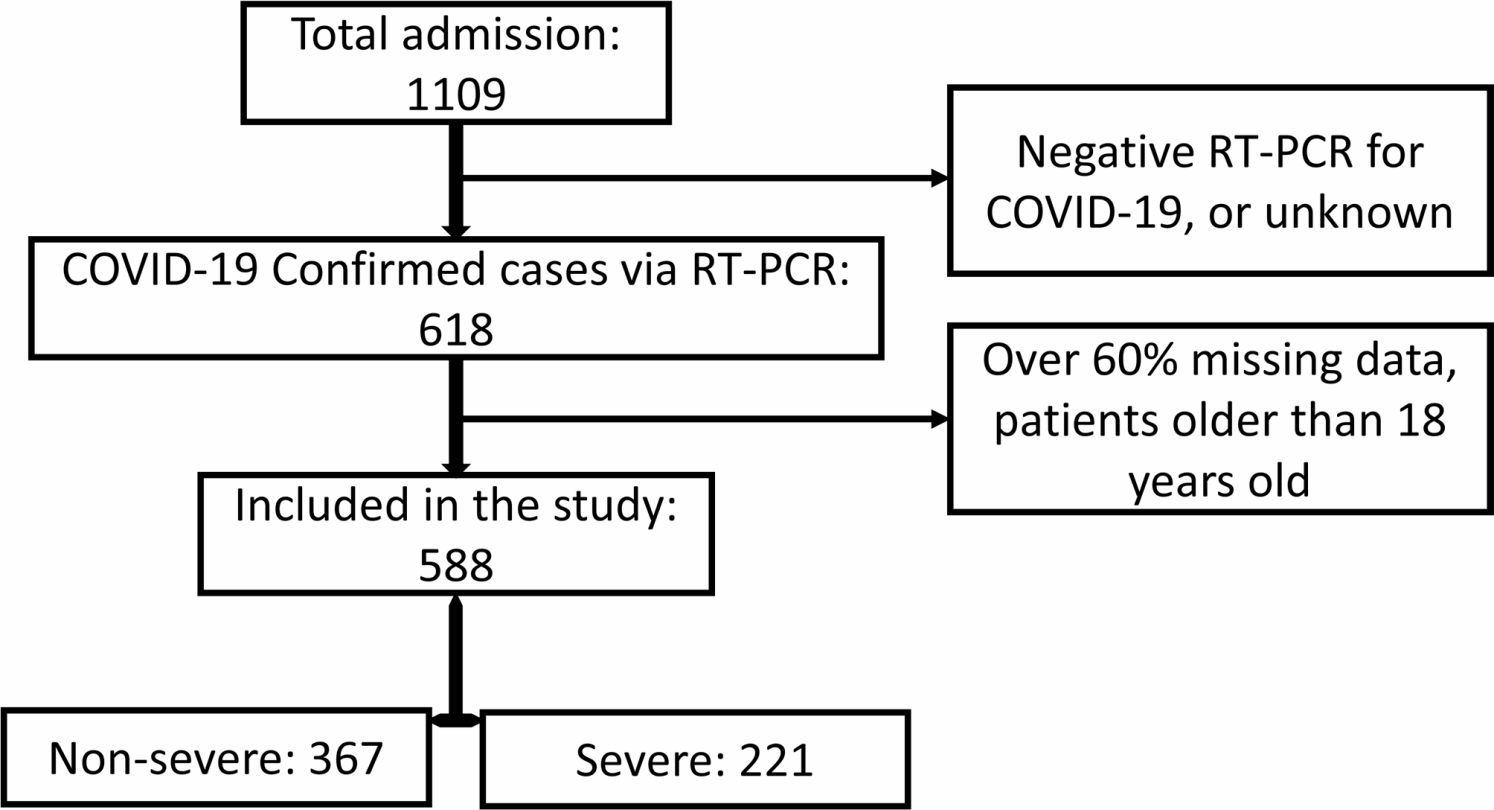
Patient selection flowchart. The flowchart details the selection process for the patient included in the study. From 1,109 initial admissions, 588 pediatric patients with confirmed COVID-19 via RT-PCR were included after applying exclusion criteria. The final patient group was divided into 367 non-severe and 221 severe cases, which were used for subsequent predictive modeling analyses.

Only positive RT-PCR COVID-19 cases were included in the study. Exclusion criteria encompassed negative RT-PCR COVID-19 test results, unknown dispositions, over 60% missing data, and patients older than 18 years old. Inclusion and exclusion criteria for the study are schematically shown in Fig. 1.

### Feature selection

To reduce the risk of overfitting in ML algorithms, selecting the key variables that have a strong correlation with the outcome variable is essential. This research, identified important variables for predicting COVID-19 severity through Generalized Boosted Models (GBM) and Random Forest (RF) models using the ‘SLScreenExtra’ package^40^. To further refine the feature set, highly correlated variables were excluded with a correlation coefficient greater than 0.75 using the ‘findCorrelation’ method from the ‘caret’ package^41^. The variables selected through this process are listed in Table 2, which can be found in the Results section. This careful selection process was designed to enhance the predictive accuracy and robustness of the models by focusing on the most relevant predictors.

### Model training and evaluation

Present research utilized several types of ML algorithms, including Neural Network (NN), Generalized Boosted Models (GBM), Random Forest (RF), Recursive Partitioning and Regression Trees (RPART), k-Nearest Neighbors (k-NN), and kernel support vector machines (KSVM), to forecast the severity of COVID-19 in children. A variety methodological approaches and their proved effectiveness in medical predictive modeling were the basis for the selection of ML algorithms for this study. Neural Networks were included due to their ability to model complex, non-linear relationships^42,43^. GBM and RF were selected for their robustness in handling high-dimensional data and their ensemble nature, which improves prediction accuracy^44,45^. RPART was chosen for its interpretability, allowing for clear decision-making processes^46^. k-NN was included due to its simplicity and effectiveness in classification tasks^47^. KSVM was selected for its powerful classification capabilities, particularly in high-dimensional spaces. It is particularly useful for binary classification tasks, such as predicting severe vs. non-severe cases^48^. By employing a diverse set of algorithms, the study aimed to leverage the unique strengths of each method, ensuring a comprehensive and robust prediction model for COVID-19 severity in children. Since the outcome variable was binary (severe vs. non-severe), all models were trained as binary classification models, optimizing performance based on metrics such as AUC.

Each model was trained using the “SuperLearner” package^49^ with specific libraries corresponding to each algorithm (e.g., SL.nnet for NN, SL.gbm for GBM), utilizing 70% of the dataset as a randomly allocated training set. The models were then evaluated on a 30% holdout set, with predictions converted to binary outcomes based on a threshold of 0.5. A 70:30 train-test split was used to evaluate all machine learning models in this study. This ratio was chosen to ensure that a sufficient amount of data was available for training while preserving a robust holdout set for performance evaluation. Given the dataset size and the relative class imbalance, alternative splits such as 80:20 or 90:10 were avoided to prevent reduced evaluation reliability and potential overfitting. Furthermore, the SuperLearner ensemble employs internal 10-fold cross-validation, which inherently supports robust performance estimation and generalization. Confusion matrices were used to assess the performance of each model, providing metrics such as accuracy, sensitivity, and specificity. Hyperparameter tuning was not performed for individual machine learning models, and default parameters were used for both the base learners and the SuperLearner ensemble. The SuperLearner framework dynamically optimizes model weights, reducing the need for manual tuning while ensuring that the ensemble effectively leverages the predictive strengths of its components. Given the dataset size, this approach helps prevent unnecessary complexity and potential overfitting. The default parameters used for each individual algorithm and the SuperLearner ensemble in this study are detailed in Supplementary Table 4. Also, to address the moderate class imbalance (62% non-severe vs. 38% severe), we considered potential effects on classifier sensitivity and specificity. However, no resampling strategies (e.g., oversampling, SMOTE, undersampling) or class weighting adjustments were applied during model training. This decision was informed by two factors: (1) the imbalance was not extreme, and (2) performance metrics such as sensitivity, specificity, and AUC remained well-balanced across models. Importantly, ensemble modeling via SuperLearner with internal cross-validation was used to further mitigate the impact of class imbalance and enhance generalizability. Nonetheless, future studies may benefit from applying and comparing resampling or cost-sensitive learning techniques to explore their potential performance gains, particularly in more imbalanced settings.

#### Mathematical overview and terminologies of machine learning models

To improve transparency, this subsection provides the fundamental formulations and terminologies for the machine learning models used in this study:

Random Forest (RF).

RF is an ensemble of decision trees trained on bootstrapped subsets of the data. The prediction for classification is given by:

ŷ = mode(T₁(x), T₂(x), …, Tₙ(x)).

where T_i_(x) is the prediction from the i-th decision tree.

Gradient Boosted Machines (GBM).

GBM builds trees sequentially, where each new tree minimizes the residual error of the previous ensemble. The model prediction is given by:

where hₘ(x_i_) is the prediction from the m-th tree, and γₘ is the step-size (often scaled by the learning rate).

Support Vector Machines (SVM).

SVM seeks to find a hyperplane that maximizes the margin between classes. The decision function is:

where α_i_ are support vector coefficients, y_i_ are class labels, K(x_i_, x) is a kernel function (e.g., RBF), and b is the bias term.

Neural Network (NN).

A feedforward neural network computes predictions as:

ŷ = f(W₂·σ(W₁·x + b₁) + b₂).

where W₁, W₂ are weight matrices, b₁, b₂ are biases, σ is an activation function (e.g., ReLU, sigmoid), and f is the final output function (e.g., softmax).

SuperLearner (SL) Ensemble.

The SuperLearner model combines multiple base learners through weighted averaging:

ŷ_SL=Σj=1Jαjŷj(x)

where ŷ_j_(x) is the prediction from the j-th base learner, and α_j_ is the optimized weight assigned to each learner.

Unlike fixed ensemble strategies such as bagging (e.g., Random Forest) or boosting (e.g., GBM), the SuperLearner framework implements a stacking-based approach, using cross-validation to learn the optimal weighted combination of diverse base learners. This method allows the ensemble to adaptively exploit the strengths of each algorithm, improving overall predictive performance and generalizability.

Each model’s mathematical foundation enhances interpretability, particularly when combined with feature importance or XAI analysis. These models were implemented using default parameters (see Supplementary Table 4), and trained on a 70/30 split of the dataset.

### Cross-Validated superlearner (CV.SuperLearner)

The CV.SuperLearner algorithm was employed to enhance predictive performance by leveraging the strengths of individual models. This ensemble method combined multiple algorithms, including NN, RF, GBM, RPART, k-NN, and KSVM. The ensemble model was trained using a 10-fold cross-validation approach to estimate the risk and compare the performance of the different algorithms. Risk estimates from the CV.SuperLearner model were summarized and visualized, offering insights into the distribution of the best single learner across external cross-validation folds. Supplementary Table 5 shows summary of machine learning models used in this study and included in the ensemble model.

Data analysis and model training were conducted using R version 4.4.1^50^ensuring robust and reproducible results through a well-established statistical computing environment.

## Results

A total of 1109 suspected COVID-19 cases were documented in the hospital’s ambulatory and emergency departments. Among these, 618 were confirmed positive for COVID-19 via RT-PCR, while 491 tested negative or unknown. Also, after applying exclusion criteria, the final sample size consisted of 588 patients, with 367 in the non-severe group and 221 in the severe group.

### Feature selection

To ensure robust model performance and minimize over-fitting, a feature selection process was implemented. A combination of 50 key variables was found to be the most significant predictors of the severity of COVID-19 in pediatric patients. The selection procedure incorporates the use of GBM and RF models, followed by additional refinement by excluding highly correlated variables. The final set of variables, detailed in Table 2, includes demographic factors, laboratory results, and clinical signs. This curated set of predictors formed the basis for training the machine learning models discussed in subsequent sections.

**Table 2:** List of variables selected for predicting COVID-19 severity in pediatric patients.

No.	Variable	Category	Type	No.	Variable	Category	Type
1	Age	Demographic	Continuous	26	Troponin_I	Laboratory	Continuous
2	Blood_Group_A	Demographic	Nominal	27	Fibrinogen	Laboratory	Continuous
3	Blood_Group_AB	Demographic	Nominal	28	Albumin	Laboratory	Continuous
4	Blood_Group_O	Demographic	Nominal	29	Proteinuria	Laboratory	Nominal
5	Sex	Demographic	Nominal	30	Hematuria	Laboratory	Nominal
6	IgG	Laboratory	Continuous	31	PCO2	Laboratory	Continuous
7	WBC	Laboratory	Continuous	32	PO2	Laboratory	Continuous
8	RBC	Laboratory	Continuous	33	HCO3	Laboratory	Continuous
9	Hemoglobin	Laboratory	Continuous	34	Chest_CT_Score	Laboratory	Continuous
10	Platelets	Laboratory	Continuous	35	Underlying_Disease	Clinical	Nominal
11	Neutrophils	Laboratory	Continuous	36	Asthma	Clinical	Nominal
12	Lymphocytes	Laboratory	Continuous	37	LVEF	Clinical	Continuous
13	Creatinine	Laboratory	Continuous	38	Valvulitis	Clinical	Nominal
14	CPK	Laboratory	Continuous	39	Coronary_Dilation	Clinical	Nominal
15	LDH	Laboratory	Continuous	40	Hypotension	Clinical	Nominal
16	Calcium	Laboratory	Continuous	41	Fever_Before_Hospitalization	Clinical	Nominal
17	Phosphorus	Laboratory	Continuous	42	Cough	Clinical	Nominal
18	Magnesium	Laboratory	Continuous	43	Conjunctivitis	Clinical	Nominal
19	Potassium	Laboratory	Continuous	44	Confusion	Clinical	Nominal
20	SGPT	Laboratory	Continuous	45	Colitis	Clinical	Nominal
21	Prothrombin_Time	Laboratory	Continuous	46	Splenomegaly	Clinical	Nominal
22	Partial_Thromboplastin_Time	Laboratory	Continuous	47	Tachypnea	Clinical	Nominal
23	CRP	Laboratory	Continuous	48	Abdominal_Pain	Clinical	Nominal
24	CD8	Laboratory	Continuous	49	O2_Saturation	Clinical	Continuous
25	Vitamin_D	Laboratory	Continuous	50	Distress	Clinical	Nominal

The table lists the variables used in the analysis, categorized by demographic, laboratory, and clinical data. *WBC* White Blood Cells, *RBC *Red Blood Cells, *CPK *Creatine Phosphokinase, *LDH *Lactate Dehydrogenase, *SGPT *Serum Glutamate Pyruvate Transaminase, *CRP *C-Reactive Protein, *CD8 *Cluster of Differentiation 8, *PCO2 *Partial Pressure of Carbon Dioxide, *PO2 *Partial Pressure of Oxygen, *HCO3 *Bicarbonate concentration, *CT Score *Computed Tomography Score, *LVEF *Left Ventricular Ejection Fraction, *O2 Saturation *Oxygen Saturation.

### Predictive performance of machine learning algorithms

In this research, the efficacy of six machine learning algorithms were assessed, including RF, GBM, KSVM, RPART, NN, and NN, in predicting the severity of COVID-19 in pediatric patients. To evaluate the predictive performance of these algorithms, various metrics such as sensitivity, specificity, positive predictive value (PPV), negative predictive value (NPV), precision, recall, F1 score, and accuracy were calculated.

The RF algorithm demonstrated the highest performance across most metrics, with an accuracy of 90.1%, sensitivity of 90.2%, and specificity of 90.1%. These results indicate that RF was the most effective model in distinguishing between severe and non-severe COVID-19 cases (Table 3). The high PPV and NPV values (91.5% and 88.5%, respectively) further highlight the robustness of RF in predicting true positive and true negative cases.

**Fig. 2:**
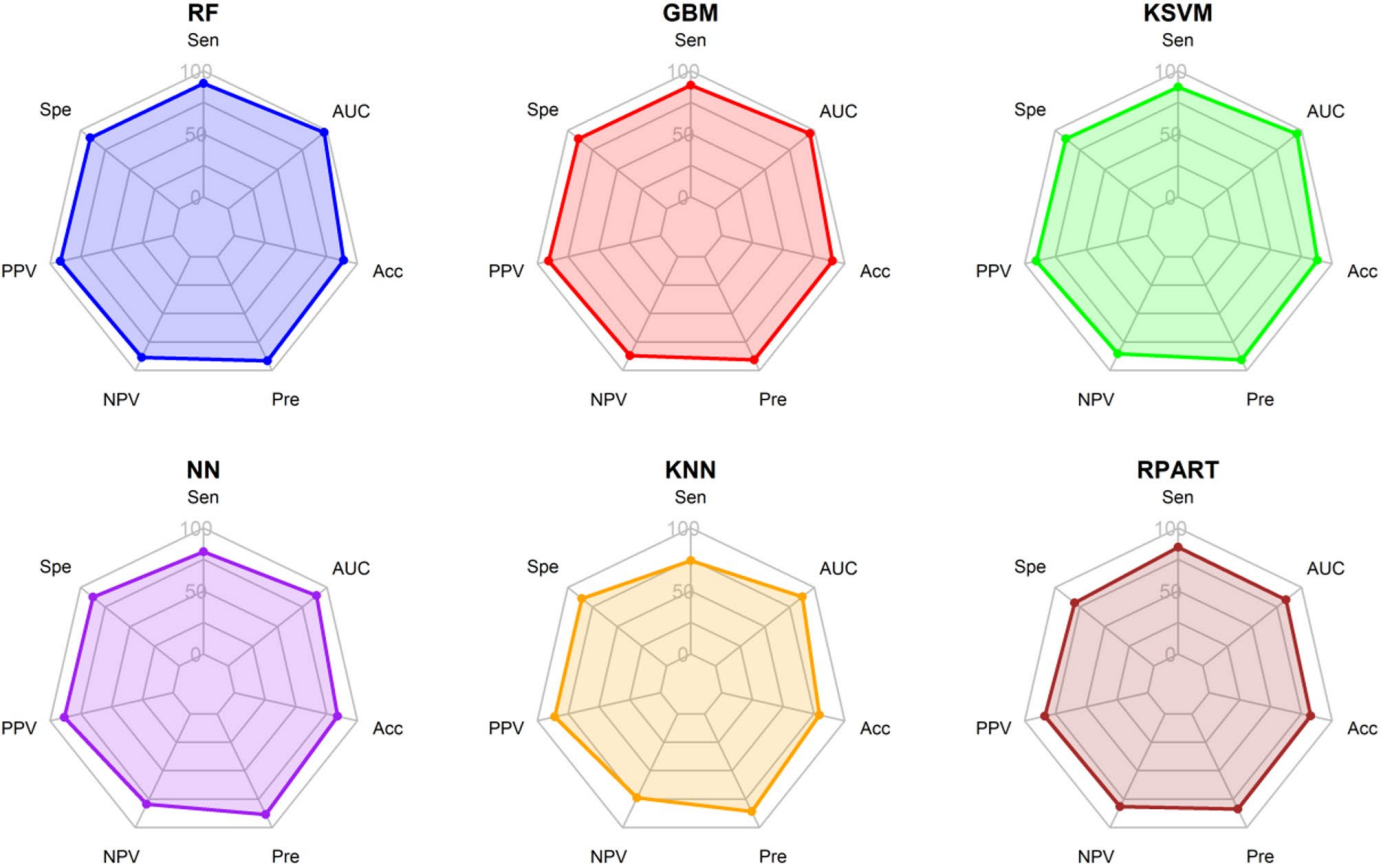
Radar plots of performance metrics for six machine learning algorithms in predicting COVID-19 severity: Random Forest (RF), Generalized Boosted Models (GBM), Kernel Support Vector Machines (KSVM), Neural Networks (NN), k-Nearest Neighbors (k-NN), and Recursive Partitioning and Regression Trees (RPART). The metrics visualized include Sensitivity (Sen), Specificity (Spe), Positive Predictive Value (PPV), Negative Predictive Value (NPV), Precision (Pre), Area Under Curve (AUC), and Accuracy (Acc).

GBM and KSVM also performed well, with accuracies of 88.9% and 88.1%, respectively. Both models showed balanced sensitivity and specificity, underscoring their reliability in clinical prediction tasks. In contrast, NN and k-NN exhibited lower performance, with NN achieving an accuracy of 84.0% and k-NN an accuracy of 79.4%. These results suggest that while NN and k-NN can still provide valuable predictions, their performance is inferior to that of RF, GBM, and KSVM (Table 3; Fig. 2).

**Fig. 3:**
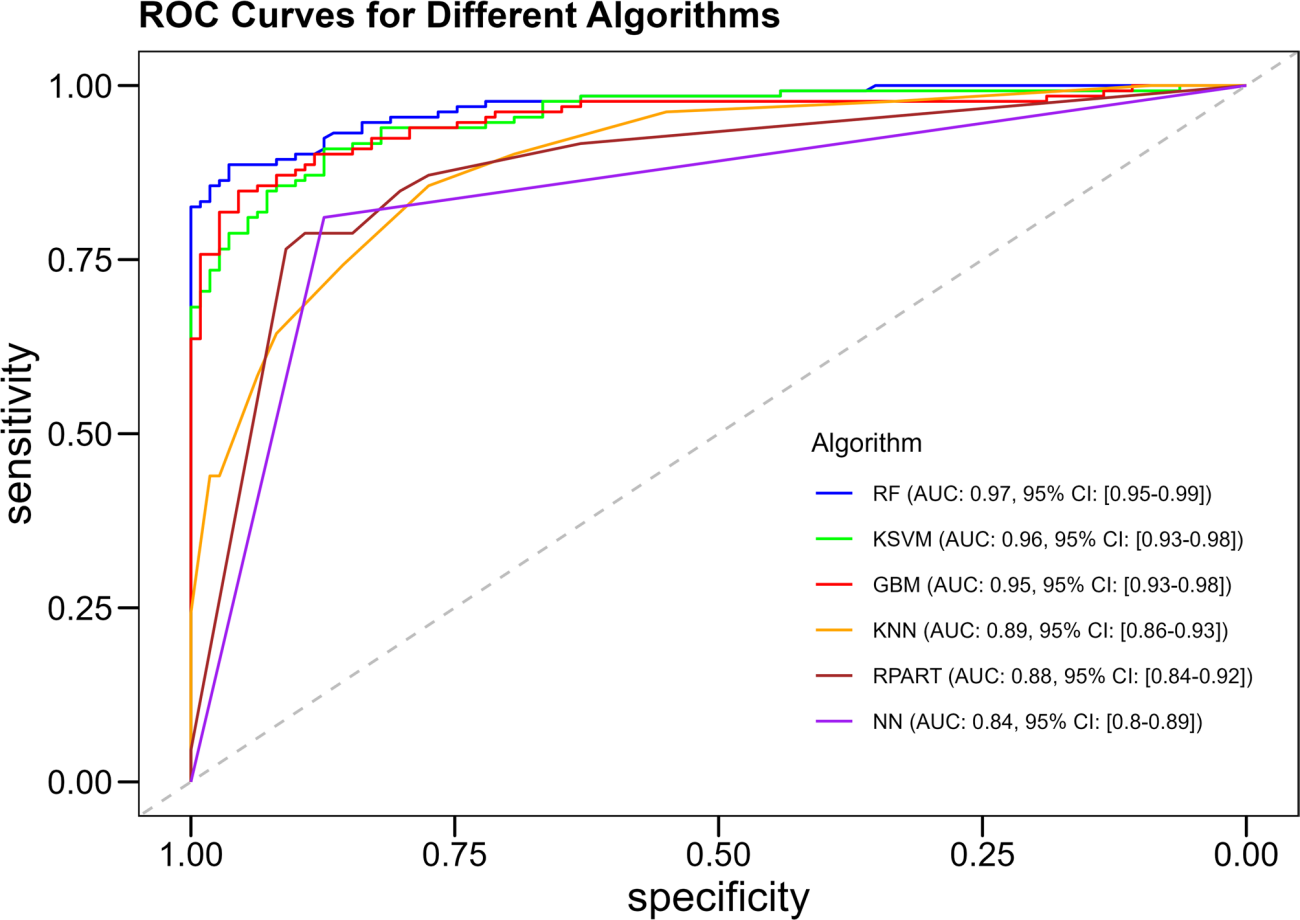
Receiver Operating Characteristic (ROC) curves for different machine learning algorithms. The ROC curves illustrate the predictive performance of various algorithms, with the Area Under Curve (AUC) indicating the overall model accuracy. Abbreviations: RF = Random Forest, KSVM = Kernel Support Vector Machine, GBM = Gradient Boosting Machine, KNN = K-Nearest Neighbors, RPART = Recursive Partitioning and Regression Trees, NN = Neural Network, AUC = Area Under Curve, CI = Confidence Interval.

The ROC curve analysis further supported these findings, with RF achieving the highest AUC of 0.972 (95% CI: 0.954–0.989), indicating excellent discriminatory ability (Fig. 1). KSVM and GBM also demonstrated strong performance with AUCs of 0.955 and 0.953, respectively. The lower AUCs for k-NN (0.894) and NN (0.842) reflect their reduced efficacy in this context (Fig. 3).

**Table 3:** Performance metrics of machine learning algorithms for predicting COVID-19 severity in pediatric patients.

Algorithm	Sensitivity	Specificity	PPV	NPV	Precision	Recall	F1 score	Accuracy
RF	0.902	0.901	0.915	0.885	0.915	0.902	0.908	0.901
GBM	0.886	0.892	0.907	0.868	0.907	0.886	0.897	0.889
KSVM	0.871	0.892	0.906	0.853	0.906	0.871	0.888	0.881
RPART	0.848	0.802	0.836	0.817	0.836	0.848	0.842	0.827
NN	0.811	0.874	0.884	0.795	0.884	0.811	0.846	0.840
k-NN	0.742	0.856	0.860	0.736	0.860	0.742	0.797	0.794

This table presents the performance metrics of six machine learning algorithms evaluated in the study: Random Forest (RF), Generalized Boosted Models (GBM), Kernel Support Vector Machines (KSVM), Recursive Partitioning and Regression Trees (RPART), Neural Networks (NN), and k-Nearest Neighbors (k-NN). The metrics include Sensitivity, Specificity, Positive Predictive Value (PPV), Negative Predictive Value (NPV), Precision, Recall, F1 Score, and Accuracy.

### Ensemble model performance

The Cross-Validated SuperLearner (CV.SuperLearner) algorithm was employed to leverage the strengths of individual models through ensemble learning. The CV.SuperLearner combined multiple algorithms, including NN, RF, GBM, RPART, k-NN, and KSVM, using a 10-fold cross-validation approach to estimate risk and compare performance.

**Fig. 4:**
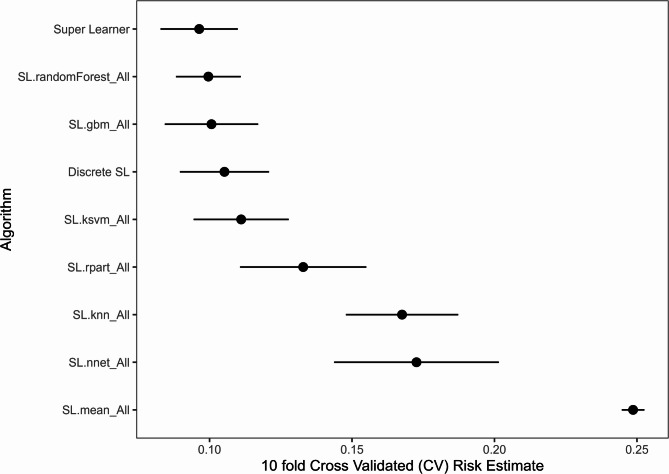
10-fold Cross-Validated (CV) Risk Estimates for various algorithms using the SuperLearner framework. This plot illustrates the average CV risk estimates with standard error bars for different algorithms, including the Super Learner ensemble model, the discrete winner, and the benchmark algorithm. Super Learner is an ensemble method that combines multiple algorithms to minimize prediction error by creating an optimal weighted average. Discrete SL (Discrete Super Learner) indicates the best-performing single algorithm based on cross-validated risk. SL.mean_All (mean prediction algorithm) is a simple mean of the response variable predictions, included as a benchmark to ensure that more sophisticated models outperform a basic mean-based prediction. The results demonstrate that the Super Learner model achieves lower risk compared to individual algorithms, confirming its advantage in ensemble learning over individual algorithms. Abbreviations used: SL.randomForest_All (Random Forest algorithm), SL.gbm_All (Gradient Boosting Machine algorithm), SL.ksvm_All (Kernel Support Vector Machine algorithm), SL.rpart_All (Recursive Partitioning and Regression Trees algorithm), SL.knn_All (k-Nearest Neighbors algorithm), SL.nnet_All (Neural Network algorithm), SL.mean_All (Simple mean prediction as a benchmark algorithm), CV (Cross-Validation).

The ensemble model exhibited superior performance compared to individual learners, with a mean risk estimate significantly lower than that of the best single learner (Fig. 4). This finding underscores the advantage of ensemble methods in predictive modeling, particularly in complex clinical datasets where individual models may capture different aspects of the data. The reduced variance in risk estimates across cross-validation folds further emphasizes the stability and reliability of the ensemble approach.

### Variable importance

Understanding the variables that most significantly influence the severity of COVID-19 in pediatric patients is crucial for both clinical practice and the development of predictive models. This study used RF and GBM to identify the key predictors of disease severity.

**Fig. 5:**
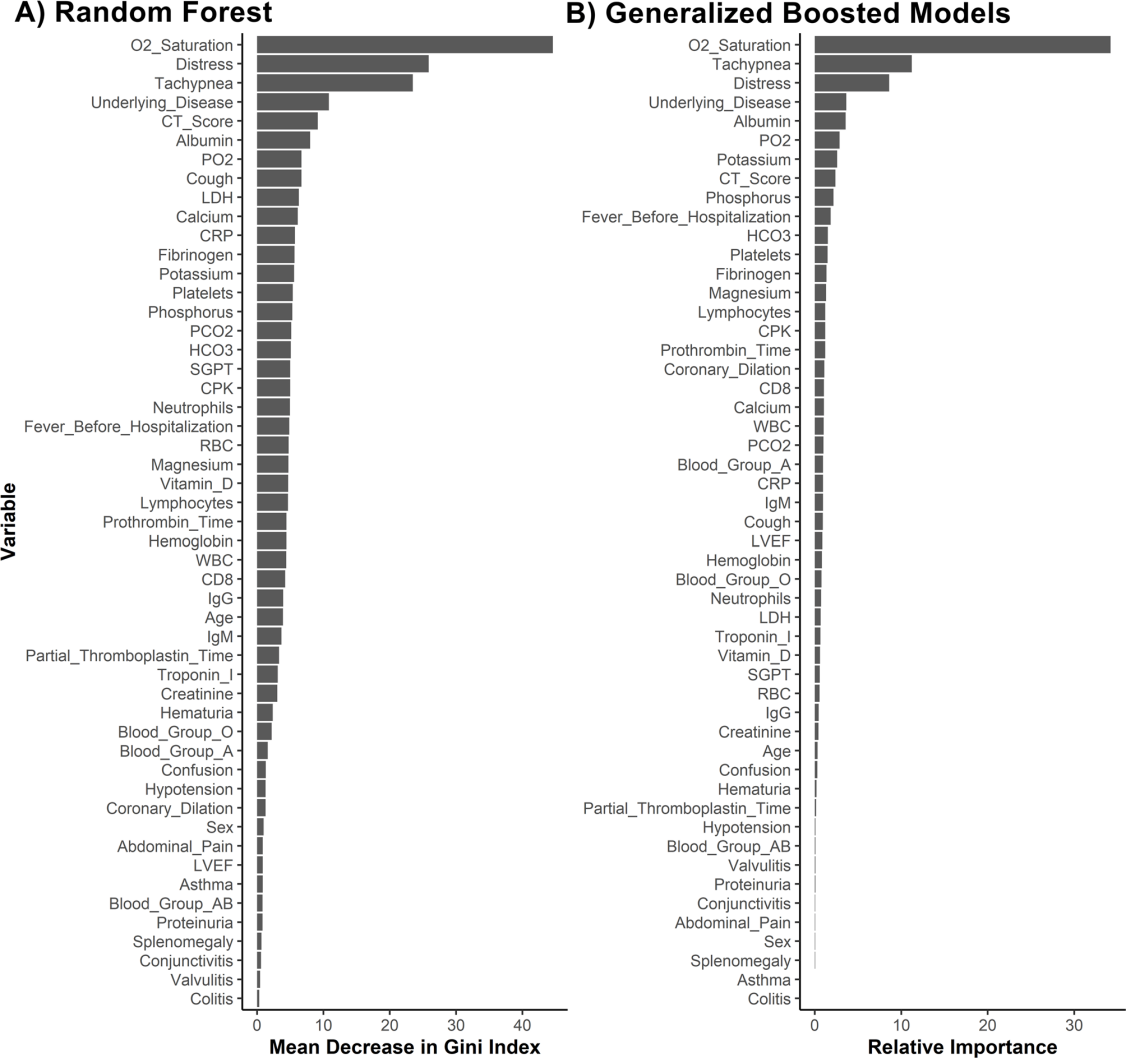
Variable importance in predicting COVID-19 severity using (**A**) Random Forest and (**B**) Generalized Boosted Models. This figure displays the importance of various clinical, demographic, and laboratory variables in predicting the severity of COVID-19 in pediatric patients, as determined by RF and GBM. The analysis highlights critical predictors, such as respiratory parameters (e.g., O2 saturation), underlying diseases, and albumin levels, which consistently rank high in both models. This visual representation underscores the relevance of specific features in the predictive modeling process, aiding in the identification of key factors that contribute to disease severity and informing targeted clinical interventions.

As shown in Fig. 5 both RF and GBM models highlighted the critical role of respiratory parameters (O2 saturation, tachypnea, and distress) in predicting COVID-19 severity in pediatric patients. The consistency in identifying O2 saturation as the top predictor underscores its importance in clinical assessments and triaging of patients.

Underlying diseases and albumin levels were significant across both models, indicating the exacerbating effect of comorbidities and the impact of nutritional and inflammatory status on disease progression. The importance of CT scores in both models highlights the value of imaging in understanding the extent of lung involvement.

While there were some differences in the specific laboratory values identified by each model (e.g., phosphorus in GBM vs. LDH and calcium in RF), the overall patterns suggest that a combination of clinical signs, laboratory markers, and imaging findings provides a comprehensive picture of disease severity.

### Explainable AI analysis

SHAP analysis was performed to assess the global contribution of each feature in the Random Forest model. Table 4 lists the top five predictors ranked by SHAP importance values. Oxygen saturation was the most influential variable, followed by respiratory indicators such as tachypnea and distress. These findings are consistent with established clinical indicators for COVID-19 severity in pediatric patients.

**Table 4:** Top 5 features ranked by SHAP importance from the random forest model.

Rank	Feature	SHAP importance
1	O₂ saturation	0.1007
2	Tachypnea	0.0071
3	LDH	0.0018
4	Albumin	0.0018
5	Distress	0.0018

## Discussion

This study assessed the performance of several machine learning (ML) algorithms in predicting COVID-19 severity among pediatric patients—a group that has received comparatively limited attention in prior predictive modeling studies. By applying an ensemble approach using the Cross-Validated SuperLearner alongside individual models such as Random Forest (RF), the analysis demonstrated that these methods can effectively differentiate between severe and non-severe pediatric cases. The integration of multiple interpretable ML models tailored to pediatric clinical data represents a meaningful contribution to improving early risk stratification in this population.

The RF model emerged as the most effective algorithm, achieving an accuracy of 90.1%, sensitivity of 90.2%, and specificity of 90.1%. This performance can be attributed to the RF model’s ability to handle high-dimensional data effectively and manage class imbalances, which are common challenges in clinical datasets. The model’s high PPV and NPV further underscore its reliability in correctly identifying both severe and non-severe cases, making it a valuable tool in pediatric COVID-19 prognosis.

In addition to RF, other models such as GBM and KSVM also demonstrated strong performance, with accuracies of 88.9% and 88.1%, respectively. The consistent performance across multiple metrics indicates these models’ robustness and their potential utility in clinical settings. GBM, known for capturing non-linear relationships within data, and KSVM, effective in high-dimensional spaces, provided complementary strengths to the predictive modeling efforts, particularly in identifying subtle clinical patterns.

A key highlight of this study was the superior performance of the CV.SuperLearner ensemble model. By combining the strengths of various algorithms, the ensemble approach significantly reduced the mean risk estimate, outperforming individual models. This finding underscores the power of ensemble learning in clinical prediction tasks, where the complexity of data often requires the integration of multiple analytical perspectives. The ensemble model’s reduced variance in risk estimates across cross-validation folds further supports its stability and reliability, making it a promising candidate for integration into clinical decision-making processes.

However, when comparing these findings with those from other studies—particularly those focused on adult populations—several differences emerge, which can be attributed to various factors. The distinct physiological and immunological characteristics of children, as well as differences in disease progression, contribute significantly to these discrepancies. For example, adult studies often emphasize comorbidities like hypertension, diabetes, and obesity as key predictors of severe COVID-19 outcomes^51–54^while this study identified respiratory parameters, such as tachypnea, and O2 saturation, as critical predictors in pediatric cases. This divergence reflects the unique clinical presentation of COVID-19 in children, who, unlike adults, are more prone to developing conditions such as multisystem inflammatory syndrome in children (MIS-C)^28^.

Differences in the types of data and features used across studies also contribute to the observed discrepancies. While adult-focused models often incorporate variables related to cardiovascular and renal health^55–57^present study prioritized laboratory markers and clinical features more relevant to pediatric patients. Additionally, the reliance on different types of imaging data across studies could influence model effectiveness^58,59^. For instance, some adult studies heavily emphasize chest CT scans, while this study integrated a broader set of biomarkers, reflecting the distinct diagnostic approaches needed for children.

SHAP analysis of the Random Forest model identified oxygen saturation as the most influential predictor of COVID-19 severity, far surpassing other variables. This finding aligns with clinical expectations, as hypoxemia is a well-established marker of respiratory compromise in pediatric patients. Other key predictors—tachypnea, LDH, albumin, and respiratory distress—also showed strong model influence and clinical relevance. Tachypnea and distress reflect early respiratory decompensation, LDH indicates tissue damage and inflammation, and hypoalbuminemia signals poor nutritional and inflammatory status. The consistency of these features across models and their alignment with known pediatric COVID-19 markers supports their use in early triage and ICU escalation. Differences in feature rankings between Random Forest and GBM are expected due to model architecture, with RF emphasizing LDH and calcium, while GBM prioritized phosphorus. Nonetheless, the top predictors remained largely consistent, reinforcing their robustness and clinical significance.

Methodological variations further explain the differences between findings from this study and those of other studies. The selection and tuning of algorithms, as well as the use of ensemble techniques like the CV.SuperLearner, can significantly impact predictive performance. While this study highlighted the effectiveness of ensemble methods in pediatric settings, other studies may have focused on single models or different ensemble strategies, leading to variations in outcomes^60–62^. Moreover, differences in study design and sample size—this study being limited to a single pediatric referral center—could affect the generalizability and variability of model performance, particularly when compared to studies involving larger, more diverse adult populations.

The implications of these divergent findings are clear: there is a critical need for developing population-specific models that can accurately capture the unique clinical characteristics of different patient groups. While adult studies provide valuable insights, their findings cannot always be directly applied to pediatric populations without significant adjustments. This study emphasizes the necessity of continued research focused on children, ensuring that predictive models are tailored to the specific needs of pediatric care.

These insights can inform clinical decision-making, enabling early identification of high-risk pediatric patients and timely interventions to improve outcomes. Future research should focus on validating these findings in larger cohorts and exploring the integration of additional biomarkers to enhance the predictive accuracy and utility of these models in diverse clinical settings.

From a computational standpoint, Gradient Boosted Machines required the longest training time due to their sequential nature, while Random Forests provided a favorable trade-off between performance and speed through parallelized training. KNN and single decision trees (e.g., RPART) were comparatively faster but less accurate. Although the SuperLearner framework introduced additional complexity by combining multiple learners through cross-validation, the resulting performance improvements justify its use. Furthermore, once trained, the ensemble can deliver predictions with minimal delay, making it feasible for clinical decision support applications in settings with sufficient computational resources.

The practical application of these predictive models lies in their potential integration into pediatric clinical workflows. For example, the models could assist emergency physicians in rapidly identifying high-risk children upon admission, thereby facilitating timely ICU referrals or escalation of care. In resource-limited settings, they may help prioritize access to diagnostics or advanced respiratory support. Given that the input features—vital signs, basic labs, and clinical observations—are routinely collected, integration into triage dashboards or electronic health record systems is feasible with minimal additional burden. These tools may be particularly valuable during public health emergencies or seasonal surges, where rapid risk stratification is critical for efficient resource allocation. Prospective studies and real-world implementation trials will be essential next steps to evaluate their clinical utility and impact on outcomes.

This study has several limitations that should be considered when interpreting the findings. First, the analysis was based on retrospective data from a single pediatric referral center, which may limit generalizability. Although internal validation demonstrated strong performance, the lack of an external validation cohort poses a risk to external validity.

A key methodological limitation is that missing data imputation was performed on the entire dataset prior to the train-test split, which may introduce a minor risk of information leakage. However, this risk is mitigated by (1) exclusion of features with > 60% missingness, (2) minimal missingness in top predictors such as oxygen saturation, LDH, and tachypnea (≤ 5%), and (3) the use of both holdout testing and ensemble-based cross-validation. Nevertheless, future studies should apply fold-wise imputation and validate findings using multicenter, prospective datasets. Incorporating real-time deployment and clinician feedback will also improve the translational value of explainable AI models in pediatric care.

Additionally, class imbalance was not explicitly addressed through resampling or weighting. While the moderate distribution (62% non-severe, 38% severe) yielded balanced performance metrics, more extreme imbalances in future or larger datasets could affect model reliability. Future research should explore techniques such as SMOTE, undersampling, or cost-sensitive learning to enhance performance in skewed settings.

Finally, feature selection was based on pairwise correlation thresholding (*r* > 0.75), which, while effective at reducing redundancy, does not fully account for higher-order multicollinearity. More advanced methods like variance inflation factor (VIF) analysis or principal component analysis (PCA) may improve statistical rigor but could compromise interpretability—a key priority in clinical machine learning. This underscores the need to balance model transparency with statistical robustness when developing decision support tools.

## Conclusion

In conclusion, this study demonstrates the value of machine learning—particularly ensemble approaches like SuperLearner—for predicting COVID-19 severity in pediatric patients. Among the models tested, ensemble learners consistently outperformed individual algorithms, with Random Forest and GBM contributing most to the ensemble’s accuracy. Explainable AI methods, including SHAP and feature importance analyses, revealed that clinical variables such as oxygen saturation and tachypnea were the most influential predictors, aligning with pediatric clinical intuition.

These findings contribute to the limited body of work focused on pediatric populations and highlight the importance of building interpretable, population-specific models for real-world clinical use. While the acute phase of the COVID-19 pandemic has subsided, such predictive frameworks remain highly relevant for pediatric triage, early intervention, and future respiratory outbreak preparedness.

The proposed models could be integrated into hospital workflows to assist clinicians with rapid risk stratification, ICU referral, and prioritization of diagnostics or monitoring resources—particularly during periods of high patient volume. Given that the model inputs are based on routinely collected clinical and laboratory data, deployment via triage dashboards or electronic health records would be feasible with minimal infrastructure. Further prospective validation and real-time implementation studies are warranted to fully realize the potential of these tools in pediatric health management.

## Supplementary Information

Below is the link to the electronic supplementary material.

Supplementary Material 1

## Data Availability

The data that support the findings of this study are available from the corresponding author upon reasonable request.

## Abbreviations

COVID-19: Coronavirus Disease 2019
ML: Machine Learning
RF: Random Forest
GBM: Gradient Boosted Machines
KSVM: Kernel Support Vector Machine
k-NN: k-Nearest Neighbors
RPART: Recursive Partitioning and Regression Trees
NN: Neural Network
SL: SuperLearner
CV: Cross-Validation
AUC: Area Under the Receiver Operating Characteristic Curve
SHAP: SHapley Additive exPlanations
CT: Computed Tomography
ICU: Intensive Care Unit
MIS-C: Multisystem Inflammatory Syndrome in Children
PPV: Positive Predictive Value
NPV: Negative Predictive Value
ROC: Receiver Operating Characteristic
O₂: Oxygen
WBC: White Blood Cells
RBC: Red Blood Cells
LDH: Lactate Dehydrogenase
CRP: C-Reactive Protein
SGPT: Serum Glutamate Pyruvate Transaminase
CD8: Cluster of Differentiation 8
LVEF: Left Ventricular Ejection Fraction
