# Supplementary material for: Predicting COVID-19 severity in pediatric patients using machine learning: a comparative analysis of algorithms and ensemble methods
Source: Sci Rep. 2025 Aug 8;15:29118. doi: 10.1038/s41598-025-15366-1 (PMC12334736; doi:10.1038/s41598-025-15366-1)
Supplement: Supplementary file 1 — Supplementary Material 1 [file 41598_2025_15366_MOESM1_ESM.pdf]

No.	Variable	Missing (%)	Unit
**Demographic Variables**			
1	Sex	0.34	Male/Female
2	Age	1.70	Years
3	BMI	41.84	kg/m^2
4	Blood_Group_A	22.62	ABO Type
5	Blood_Group_AB	22.62	ABO Type
6	Blood_Group_B	22.62	ABO Type
7	Blood_Group_O	22.62	ABO Type
**Laboratory Variables**			
8	WBC	2.21	$10^3/\mu L$
9	RBC	2.21	$10^6/\mu L$
10	Hgb	1.36	g/dL
11	Plt	1.53	$10^3/\mu L$
12	Neut	2.55	%
13	Lymph	2.89	%
14	CRP	4.93	mg/L
15	ESR	6.12	mm/hr
16	CD4	41.84	cells/µL
17	CD8	42.18	cells/µL
18	IgM	39.63	AU/mL
19	IgG	35.54	AU/mL
20	Vitamin D	48.30	ng/mL
21	CPK	18.54	U/L
22	LDH	18.88	U/L
23	CK-MB	43.88	U/L
24	TnI	51.87	ng/mL
25	IL-6	94.39	pg/mL
26	Ferritin	22.28	ng/mL
27	D-Dimer	30.10	µg/mL
28	Fibrinogen	29.59	mg/dL
29	Albumin	28.06	g/dL
30	Ca	17.18	mg/dL
31	P	20.75	mg/dL
32	Mg	19.39	mg/dL
33	Na	2.04	mmol/L
34	K	2.72	mmol/L
35	AST	12.24	U/L
36	ALT	12.41	U/L
37	PT	7.48	sec
38	PTT	7.48	sec
39	INR	7.82	Ratio
40	Proteinuria	16.67	Pos/Neg
41	Hematuria	26.53	Pos/Neg
42	BS	20.58	mg/dL
43	BUN	1.87	mg/dL
44	Cr	2.04	mg/dL

45	pH	11.90	—
46	pO2	11.05	mmHg
47	pCO2	11.22	mmHg
48	HCO3	11.22	mmol/L
**Clinical Variables**			
49	Asthma	0.34	Binary
50	UD (Underlying Disease)	18.20	Binary
51	ID (Immunodeficiency)	0.34	Binary
52	SE (Side Effects)	39.46	Binary
53	CPF (COVID-Positive Family)	4.08	Binary
54	BC (Blood Culture)	0.17	Pos/Neg
55	UC (Urine Culture)	0.17	Pos/Neg
56	LVEF	18.88	%
57	Myocarditis	15.14	Binary
58	Valvulitis	9.52	Binary
59	CorD (Coronary Dilation)	9.69	Binary
60	AKI	1.19	Binary
61	Hypotension	1.53	Binary
62	Fever	1.19	Binary
63	Fever Before Hospitalization	10.37	Binary
64	CT Score	56.63	Score
65	Lymphadenopathy	0.00	Binary
66	Lymphadenitis	0.51	Binary
67	Arthritis/Arthralgia	0.34	Binary
68	Hand Foot Edema	0.17	Binary
69	Neck Swelling	0.17	Binary
70	Syncope	0.17	Binary
71	Ileitis	3.40	Binary
72	Ascites	3.57	Binary
73	Colitis	4.76	Binary
74	Splenomegaly	4.08	Binary
75	Hepatomegaly	4.08	Binary
76	Pleural Effusion	4.59	Binary
77	O2 Saturation	5.10	%
78	O2Need	0.85	Binary
79	Distress	0.68	Binary
80	Cough	2.38	Binary
81	Chest Pain	0.34	Binary
82	Tachypnea	0.17	Binary
83	Seizure	3.06	Binary
84	Headache	0.34	Binary
85	Vomiting	0.00	Binary
86	Abdominal Pain	0.17	Binary
87	Diarrhea	0.17	Binary
88	Rhinorrhea	0.17	Binary
89	Sore Throat	1.53	Binary
90	Rash	0.34	Binary
91	Conj (Conjunctivitis)	0.51	Binary

92	Confusion	0.51	Binary
93	Myalgia	0.34	Binary

Supplementary table 1. Categorized list of all 93 variables included in the analysis, grouped into Demographic, Laboratory, and Clinical categories. The table reports each variable's name, percentage of missing values, and associated measurement unit where applicable.

Abbreviations: UD = Underlying Disease, ID = Immune Deficiency, LVEF = Left Ventricular Ejection Fraction, Vit-D = Vitamin D, CPF = COVID-Positive Family, BC = Blood Culture, UC = Urine Culture, WBC = White Blood Cell, RBC = Red Blood Cell, SE = Side Effects, BS = Blood Sugar, BUN = Blood Urea Nitrogen, Cr = Creatinine, CPK = Creatine Phosphokinase, LDH = Lactate Dehydrogenase, PT = Prothrombin Time, PTT = Partial Thromboplastin Time, INR = International Normalized Ratio, CRP = C-Reactive Protein, ESR = Erythrocyte Sedimentation Rate, CK-MB = Creatine Kinase MB, TnI = Troponin I, IL-6 = Interleukin 6, Fib = Fibrinogen, Alb = Albumin, CorD = Coronary Dilation, AKI = Acute Kidney Injury, BMI = Body Mass Index.

Feature Category	Number of Features	Data Type(s)	Description
Demographic	3	Nominal, Continuous	Age, sex, blood group
Clinical Characteristics	40	Nominal, Continuous	Symptoms (e.g., cough, fever), vital signs (e.g., O_2 saturation, tachypnea)
Comorbidity History	6	Nominal	Underlying diseases (e.g., asthma, cardiac conditions)
Laboratory Results	43	Continuous, Nominal	Blood chemistry, hematology, inflammatory markers, CT score
Outcome Variable	1	Binary	COVID-19 severity (0: Non-severe, 1: Severe)
Total Patients	588	—	Pediatric patients with confirmed RT-PCR-positive COVID-19
Non-severe Cases	367 (62.4%)	—	—
Severe Cases	221 (37.6%)	—	—

Supplementary Table 2. Structure and class distribution of the pediatric COVID-19 dataset.
This table summarizes the primary feature categories, number of features, and class balance (severe vs. non-severe cases) before modeling.

Attribute	Description
Dataset name	Pediatric COVID-19 Clinical Dataset
Number of samples	810 (before balancing), 1,566 (after SMOTE)
Number of features	50 selected clinical features
Target variable	COVID-19 severity (Severe vs. Non-Severe)
Classification type	Binary
Class distribution	25% Severe, 75% Non-Severe (before balancing)
Balancing method	SMOTE (Synthetic Minority Over-sampling Technique) applied to training data

Supplementary Table 3. Overview of dataset characteristics used for model training.

Includes details on data balancing (via SMOTE), final number of features, class distribution, and key modeling variables.

Algorithm	Parameter1	Parameter2	Parameter3	Parameter4	Parameter5	Parameter6	Parameter7	Parameter8	Parameter9
NN	size = 5	linout = FALSE	entropy = FALSE	softmax = FALSE	censored = FALSE	skip = FALSE	rang = 0.7	decay = 0	maxit = 100
GBM	n.trees = 100	interaction.depth = 1	shrinkage = 0.1	n.minobsinnode = 10					
RF	ntree = 500	mtry = sqrt(p)	nodesize = 1						
RPART	cp = 0.01	minsplit = 20	minbucket = 7						
k-NN	k = 10	distance = 2							
KSVM	kernel = 'rbfdot'	C = 1	epsilon = 0.1						
CV.SuperLearner	V = 10	family = gaussian()	method = 'method.NNLS'						

Supplementary Table 4. Default parameters used for individual machine learning algorithms and the SuperLearner ensemble.

Outlines the configuration of each base learner (e.g., GBM, RF, NN) and the SuperLearner meta-model settings.

For NN (Neural Network): size: Number of units in the hidden layer, linout: Linear output, entropy: Entropy regularization, softmax: Softmax output function, censored: Censored output, skip: Skip-layer connections, rang: Range of initial random weights, decay: Weight decay, maxit: Maximum number of iterations.

For GBM (Generalized Boosted Models): n.trees: Number of boosting iterations, interaction.depth: Maximum depth of variable interactions, shrinkage: Learning rate, n.minobsinnode: Minimum number of observations in trees.

For RF (Random Forest): ntree: Number of trees in the forest, mtry: Number of variables randomly sampled as candidates at each split, nodesize: Minimum size of terminal nodes.

For RPART (Recursive Partitioning and Regression Trees): cp: Complexity parameter, minsplit: Minimum number of observations required to attempt a split, minbucket: Minimum number of observations in any terminal node.

For k-NN (k-Nearest Neighbors): k: Number of nearest neighbors, distance: Distance metric.

For KSVM (Kernel Support Vector Machine): kernel: Kernel function, C: Regularization parameter, epsilon: Epsilon in the insensitive-loss function.

For CV.SuperLearner: V: Number of cross-validation folds, family: Error distribution family, method: Method to combine the predictions of base learners.

Model	Type	SuperLearner Function	Included in Ensemble
**Random Forest**	Tree-based	SL.randomForest	Yes
**Gradient Boosting Machine (GBM)**	Ensemble of trees	SL.gbm	Yes
**Neural Network**	Feedforward NN	SL.nnet	Yes
**Support Vector Machine (SVM)**	Kernel-based	SL.ksvm	Yes
**K-Nearest Neighbors (KNN)**	Instance-based	SL.knn	Yes
**RPART**	Decision Tree	SL.rpart	Yes
**Mean Learner**	Baseline Averaging	SL.mean	Yes

Supplementary Table 5. List of machine learning algorithms included in the SuperLearner ensemble.

Describes each model's type, corresponding SuperLearner function, and whether it was included in the final ensemble.
